# Study on the UV FEL single-shot damage threshold of an Au thin film

**DOI:** 10.1107/S1600577524005332

**Published:** 2024-07-23

**Authors:** Meiyi Wu, Qinming Li, Mingchang Wang, Qinghao Zhu, Jiandong Cai, Zhongmin Xu, Hao Zhou, Long Huang, Bingbing Zhang, Runze Qi, Zhe Zhang, Wenbin Li, Yinpeng Zhong, Weiqing Zhang

**Affiliations:** a Institute of Advanced Science Facilities, Shenzhen518107, People’s Republic of China; b State Key Laboratory of Molecular Reaction Dynamics, Dalian Institute of Chemical Physics Chinese Academy of Sciences Dalian116023 People’s Republic of China; c University of Chinese Academy of Sciences Beijing101408 People’s Republic of China; d MOE Key Laboratory of Advanced Micro-Structured Materials, Shanghai200092, People’s Republic of China; e Institute of Precision Optical Engineering, School of Physics Science and Engineering Tongji University Shanghai200092 People’s Republic of China; Deutsches Elektronen-Synchrotron, Germany

**Keywords:** free-electron laser, beamline optics, thin film coating, single-shot damage threshold, Au

## Abstract

The damage threshold of an Au-coated flat mirror, one of the reflective optics installed on the FEL-2 beamline of the Dalian Coherent Light Source, China, upon far-UV free-electron laser irradiation is evaluated. The surface of the coating is characterized by profilometer and optical microscope. A theoretical approach of the phenomenon is also presented, by application of conventional single-pulse damage threshold calculations, a one-dimensional thermal diffusion model, as well as finite-element analysis with *ANSYS*.

## Abstract 2

Single-shot damage of an 200 nm-thick Au thin film is experimentally studied with a UV FEL of 121.6 nm wavelength and 2 ps pulse length. The characterized, as well as visually observed, phenomena are analyzed with multiple theoretical approaches.

## 1. Introduction

As a fourth-generation advanced light source, the free-electron laser (FEL) provides an extremely high intensity photon beam with ultra-short pulses, where wavelength varies from the infrared to hard X-rays (Allaria *et al.*, 2012 ▸; Emma *et al.*, 2010 ▸). Despite being of great interest in many scientific domains, the development and advancement of FELs also face challenges, of which we focus here on damage to reflective optics.

Optics such as thin-film-coated mirrors are widely used on beam transport systems of advanced light sources like synchrotron and free-electron laser facilities. High-intensity photon beams may cause irreversible permanent damage on the surface of mirrors (Pelka *et al.*, 2009 ▸; Hau-Riege *et al.*, 2010 ▸; Aquila *et al.*, 2015 ▸; Koyama *et al.*, 2016 ▸; Milov *et al.*, 2018 ▸; Norman *et al.*, 2012 ▸; Ishino *et al.*, 2018 ▸; Juha *et al.*, 2009 ▸; Barkusky *et al.*, 2010 ▸; Li *et al.*, 2021 ▸; Pan *et al.*, 2022 ▸). Such damage may induce a negative impact on the optical performance of the optics, namely on the reflectivity and wavefront preservation. In some cases, the damage is too severe that it is necessary to re-polish, recoat or even repurchase the optics. To minimize unwanted yet possible damage, one should both select the coating for the optics and monitor carefully their working condition. For the coating selection, low-*Z* materials such as B_4_C are favorable due to their low absorption and high reflectance. Yet metal coatings like Au, Pt and Ru offer a higher critical angle leading to wider applications and more use cases. The working condition here mostly refers to the incidence fluence of the FEL beam on the optics and geometric conditions like the incidence angle between the FEL beam and the surface of the optics. The single-shot damage threshold (SSDT) may be used as a metric for the damage tolerance of the optics against the FEL irradiation. It is the energy density that describes the FEL energy absorbed by the coated thin film within a unit surface (per cm^2^, for example) that will likely cause irreversible surface damage. One must note that the multiple-shot case may lead to different conclusions concerning the damage threshold depending on the FEL repetition rate, but this is out of the scope of the discussion here, as explained later.

In this paper, we study the damage of a 200 nm Au film coated on a Si substrate irradiated by a FEL beam of 121.6 nm wavelength and 2 ps pulse length in normal incidence conditions. The selection of the wavelength is related to the application of the studied Au-coated optics. A 121.6 nm far-UV FEL beam is often used for scientific research performed at the Dalian Coherent Light Source (DCLS), China. It can efficiently excite hydrogen atoms (Xu *et al.*, 2014 ▸; Kosmala *et al.*, 2021 ▸; Yang *et al.*, 2015 ▸; Chang *et al.*, 2020 ▸; Xie *et al.*, 2020 ▸; Foot, 2005 ▸) without generating unwanted spectroscopic background. This makes it a characteristic wavelength for research domains such as catalytic intermediates, chemical reaction dynamics, interstellar chemistry, fundamental physics, *etc*. As the temporal scale of the studied physical and chemical phenomena at DCLS ranges within the picosecond regime (Zhang *et al.*, 2020 ▸; Chang *et al.*, 2021 ▸; Zhou *et al.*, 2019 ▸), the selection of a pulse length of 2 ps is also related to the various experimental requirements of the FEL beam. Different incidence pulse energies of the FEL are selected based on the theoretical single-shot damage threshold of the Au film.

## 2. Experimental setup

The sample is a 200 nm-thick Au film deposited on a 30 mm × 20 mm × 10 mm (L × W × H) Si substrate using physical vapor deposition. A 30 nm-thick Cr adhesion layer is inserted at the Au/Si interface to prevent the Au layer from peeling off; this is a spare piece of optics for one of the beamline instruments of the FEL-2 beamline at DCLS. It diagnoses the polarization of the incident FEL beam with four Au-coated mirrors that work as polarizers. The polarization state of the incident FEL is obtained by fitting the beam intensity passing through the polarizer as a function of the azimuth angle of the polarizer (Clemens *et al.*, 2017 ▸; Gaballah *et al.*, 2018 ▸).

**Figure 1:**
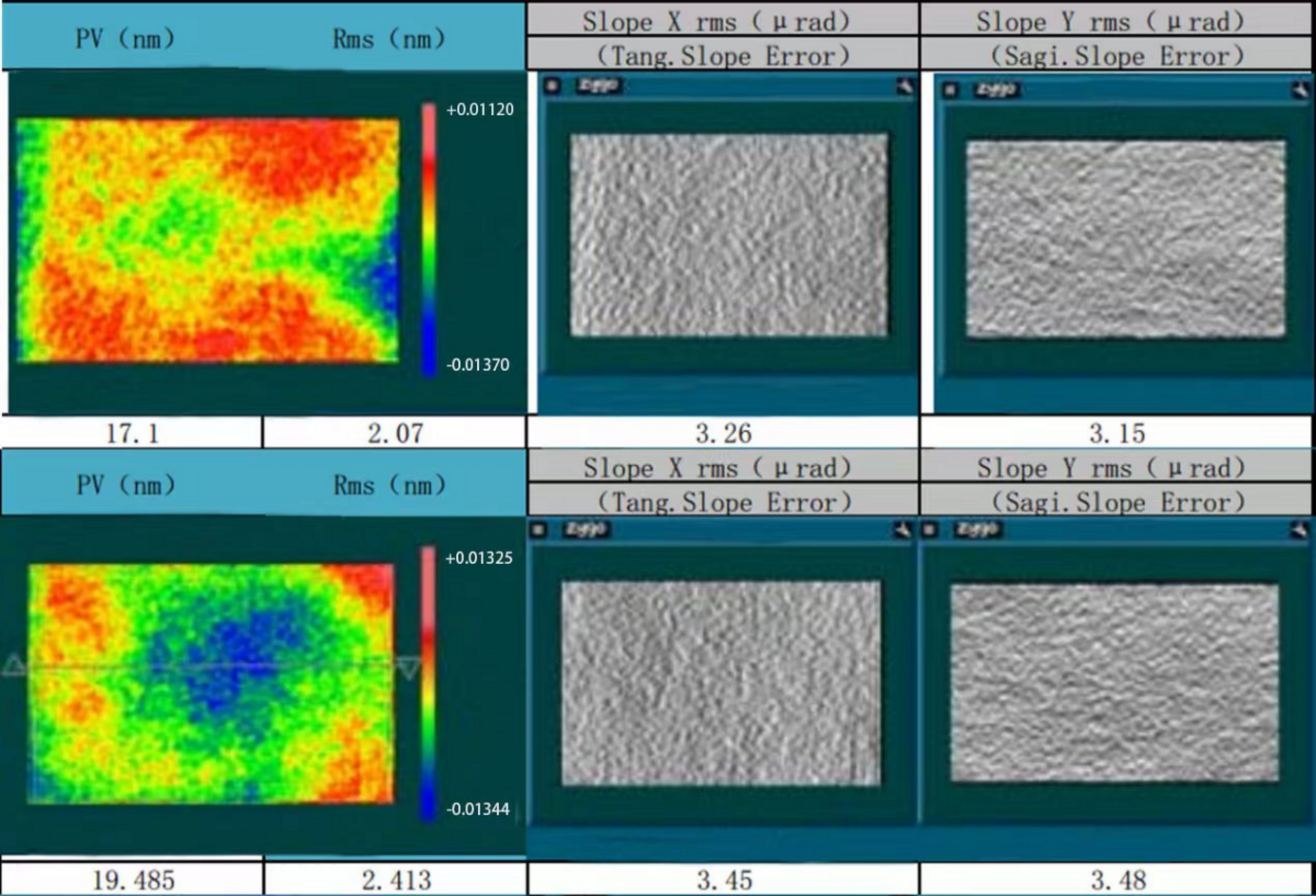
Surface quality of the sample before (top) and after (bottom) the Au/Cr deposition.

The surface quality of the sample before and after the Au/Cr deposition is characterized by Zygo’s phase-shifting Fizeau interferometry, which is widely used for precision measurements of optical wavefronts and surface profiles (de Groot, 1997 ▸, 2004 ▸; Vivo *et al.*, 2016 ▸; Huang *et al.*, 2020 ▸; Da Silva *et al.*, 2023 ▸; Shreiber & Bruning, 2007 ▸). The result is presented in Fig. 1 ▸ for a slope error of about 3 µrad. In this case the sample may also imitate a flat reflecting mirror of a regular FEL beam transport system. A slight increase of about 14% peak-to-valley (PV) is observed after the coating process. The slope error also increases accordingly by about 10%. One must note that, for other samples of the same batch, PV and slope error may also slightly decrease after the coating, despite being relatively rare cases. Therefore, we may consider that the impact of the coating on the surface quality of this kind of optics is negligible.

**Figure 2:**
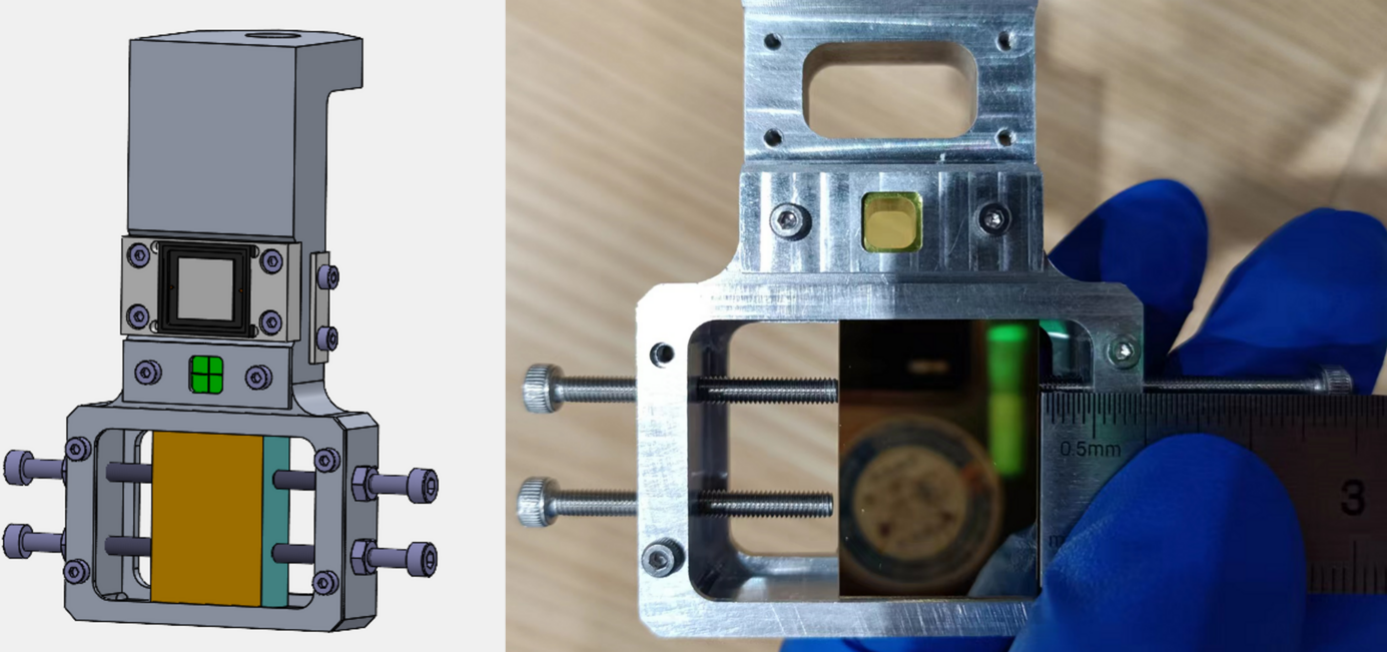
Sample holder of the experiment. Left: engineering drawing of the sample holder. Right: photograph of the manufactured sample holder with the sample and a YAG mounted on it.

The sample is mounted on a homemade vacuum-compatible holder, shown in Fig. 2 ▸. In this simple design a small piece of YAG fluorescent target is mounted above the sample to locate the beam position, and also to determine the beam spot profile. A slot above the YAG was originally left for a photodiode detector for incident beam intensity measurement (together with a solid attenuator covering the photodiode for protection purposes). Eventually a built-in intensity monitor installed at the upper stream of the beamline was used instead for determination of the incident FEL pulse energy. The intensity monitor is based on the principle of gas ionization – through the collection of ionized electrons the incident light intensity is determined according to the quantity of electric charge. The sample can move horizontally by manual adjustment with four screws, and vertically by a drive motor from outside the vacuum chamber.

**Figure 3:**
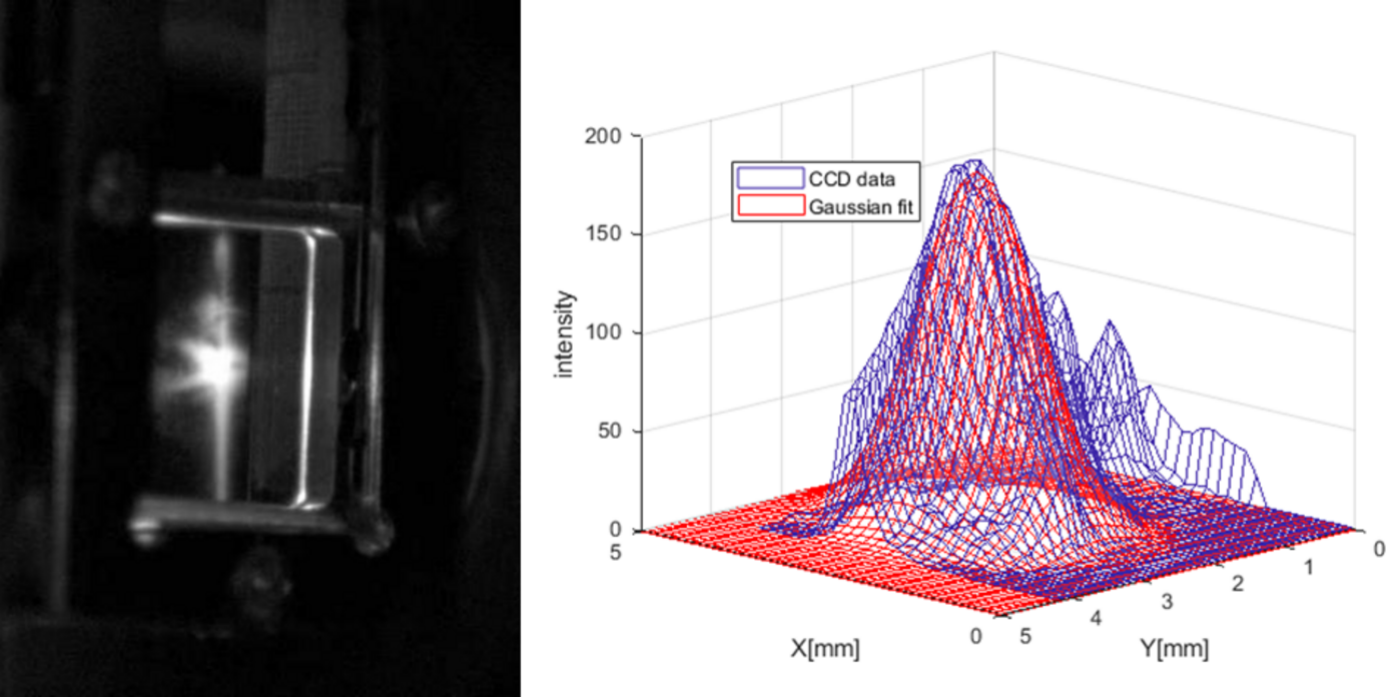
FEL beam spot profile measurement by CCD camera (left) and beam size fitting using a Gaussian (right).

For the incident FEL beam, a wavelength of 121.6 nm is selected. The general working geometry of this mirror is grazing-incidence mode. We intentionally select normal incidence exposure to enhance the damage (higher incidence energy density due to smaller footprint, more absorption resulting from lower reflectance). By comparing the normal-/grazing-incidence cases it is possible to deduce a safe working threshold of the mirror. The profile of the beam spot is roughly measured with a charge-coupled device (CCD) from outside the chamber through the observation window. An example is presented in Fig. 3 ▸. The CCD is set 45° from the sample surface. It captures images of the visible fluorescent spot size on the YAG (of 5 mm × 5 mm size) generated from FEL irradiation. From the figure it can be seen that the profile of the light spot is obviously not an ideal Gaussian, due to obvious diffraction. Beam instability may also contribute, but is not discussed here. The size of the FEL beam is determined by Gaussian fitting of the gray scale of the images of the fluorescent spot which is about 1.2 mm [full width at half-maximum (FWHM)]. The geometry of the CCD (45° from the sample surface), which causes enlargement of the fitting result, is considered in the original image acquisition in order to recover the measurement equivalent to normal-incidence observation.

## 3. Results and discussion

**Figure 4:**
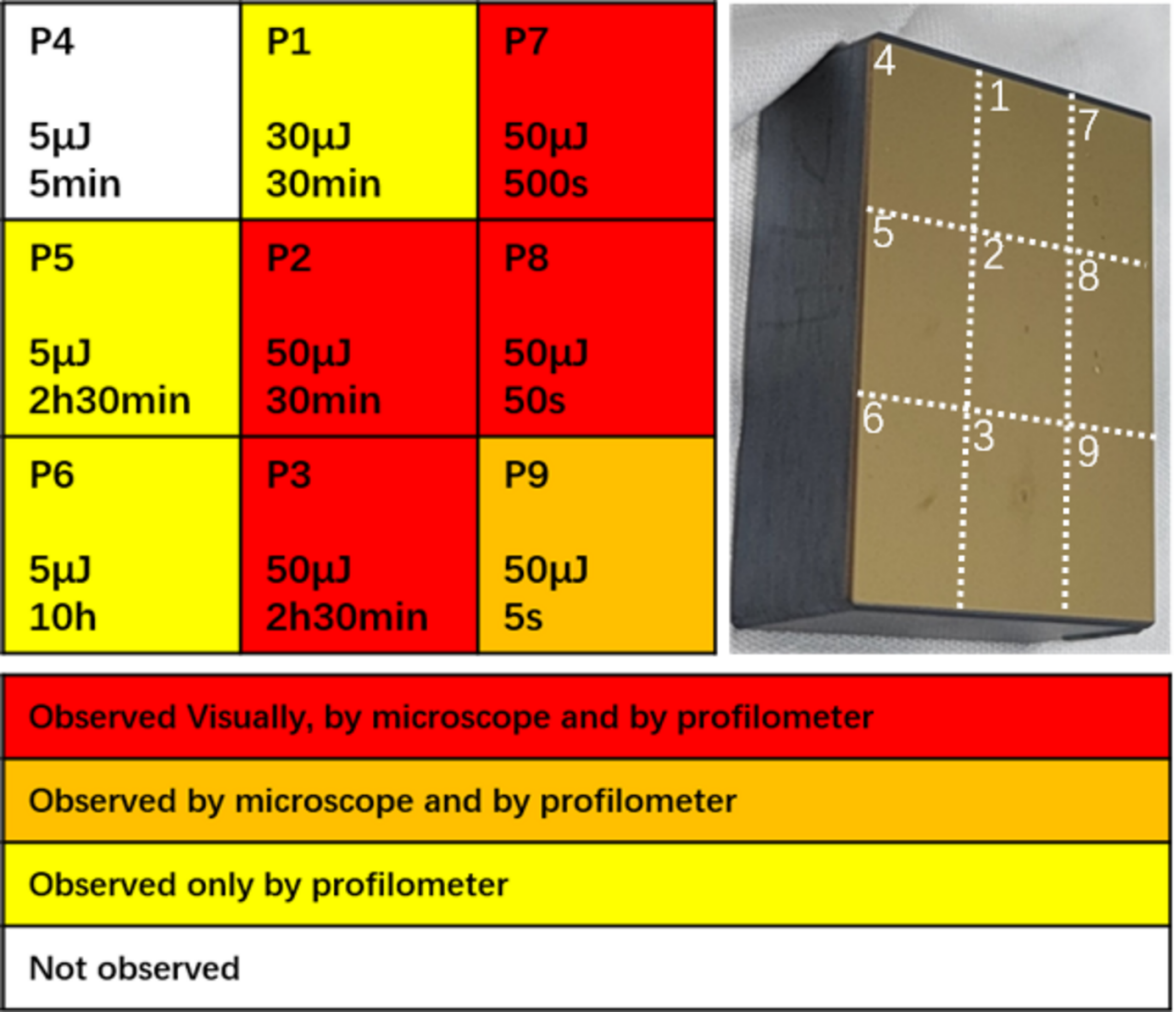
Overview of the experimental arrangement and a photograph of the Au-coated mirror after irradiation on nine points.

An overview of the experimental result is given in Fig. 4 ▸. In each cell the three lines of data indicate the following sequence: measurement position, incidence FEL pulse energy, and time of exposure, from top to bottom. The four colors of the cells represent four types of observed results: red means surface damage is visually observed, and is (naturally) also observed by optical microscope and profilometer; orange means damage is not observed by the eye but is observed by microscope and profilometer; yellow means damage is only observed by profilometer; white means nothing is observed on the irradiated position by any available means. In the photograph in Fig. 4 ▸, a darkened halo may be observed around certain irradiated positions. This is likely due to carbon deposition during the irradiation process as the vacuum level is barely 10^−6^ mbar. Details about this carbon deposition will be discussed later.

The FEL pulse energy before and after each exposure is monitored by a photodiode. The recorded pulse energy value is the normalization of an average of a continuous reading over one minute for a repetition rate of 20 Hz.

**Figure 5:**
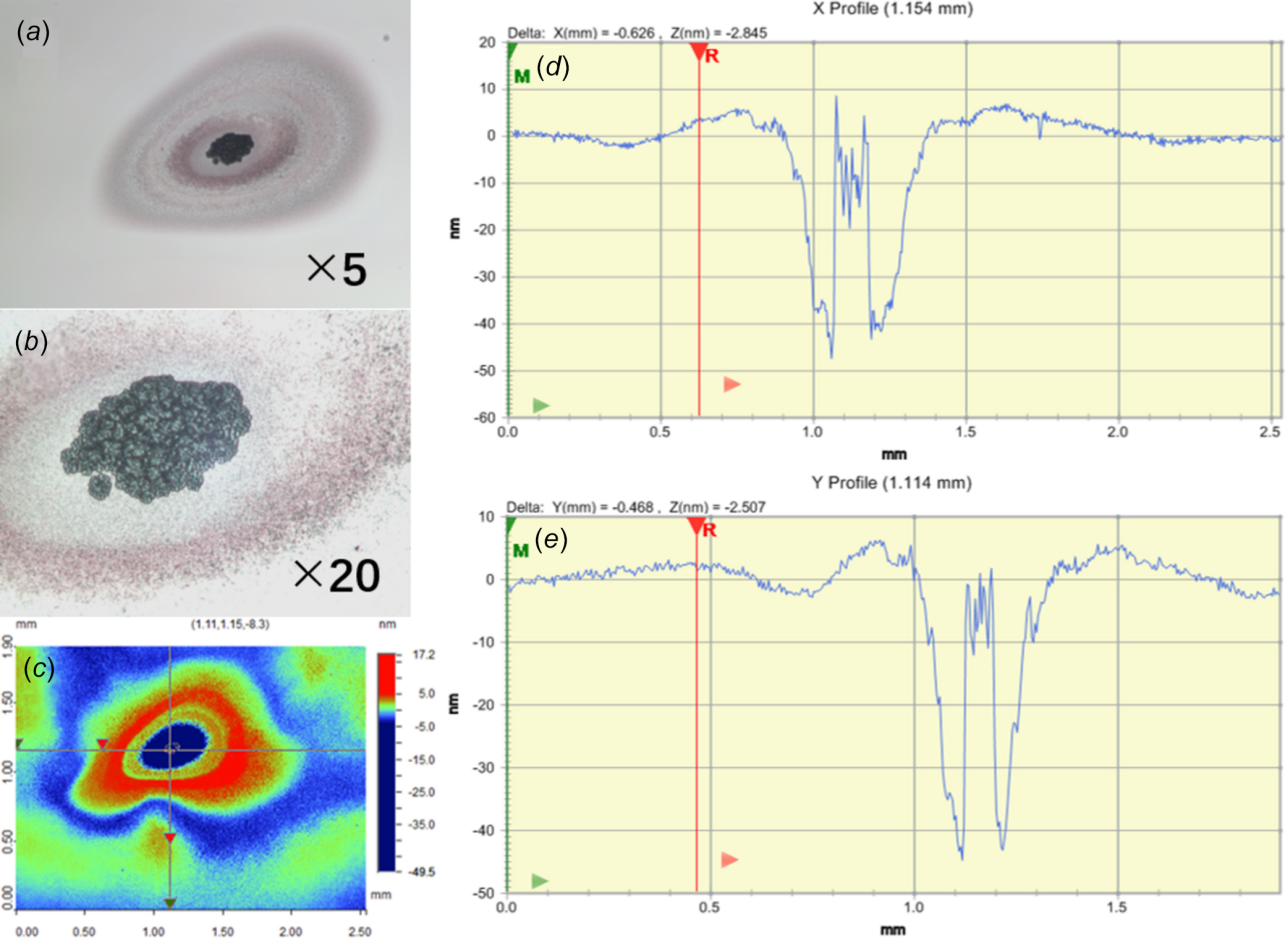
Surface characterization of the irradiated position P3 (pulse energy 50 µJ, time of exposure 2 h 30 min) including optical microscope observation with 5× (*a*) and 20× (*b*) magnification. (*c*) Profilometer-measured data with line profiles along the *x* (*d*) and *y* (*e*) axes.

For each irradiated position, whether visually observed or not, optical microscope and profilometer are applied to characterize the surface. A typical example of the acquired data is given in Fig. 5 ▸. For this particular point, the highest FEL pulse energy of 50 µJ is used with a long exposure time of 2.5 h. The crater on the surface of the thin film exhibits a shockwave resulting shape, which is typical for an ultra-fast ultra-intense beam–matter interaction with pico- and femtosecond pulse length (Aquila *et al.*, 2015 ▸; Koyama *et al.*, 2016 ▸; Milov *et al.*, 2018 ▸; Norman *et al.*, 2012 ▸; Ishino *et al.*, 2018 ▸; Barkusky *et al.*, 2010 ▸). From the first glimpse of the image taken by the optical microscope, one may easily misunderstand the high contrast between the crater center and undamaged area as evidence of the damage penetrating the entire layer all the way down to the substrate. But this is not in accordance with the fact that the Au film is 200 nm thick while the depth profiles of this crater along the *X* and *Y* axes of the surface shows that the crater is merely 40 nm deep. A reasonable explanation of this contrast would be the tremendously increased roughness of the irradiated crater center, which endures the majority of the incoming energy of the Gaussian-like distributed FEL beam. The center rises like a tower from a basin following hydro­dynamic rules, in analogy with a water drop causing bounce-back of an originally calm liquid surface, as the damaged area is in the Au liquid phase during the damaging process. The increase of the roughness has two possible interpretations. First, the ultrafast bombardment generates Au droplets or clusters which take off from the irradiated surface; such material loss is related to the beam intensity distribution, but it also possesses its own randomness. Second, the previously mentioned carbon deposition due to surface contamination may also play a role here. During the 2.5 h of FEL exposure, Au removal–redeposition and carbon deposition repeatedly occurs on the surface. The high-roughness area can be a physical mixture of Au and carbon. This deduction can be verified by performing comparative X-ray photoelectron spectroscopic measurements of carbon 1*s* spectra on both irradiated center and unirradiated area. Unfortunately, this is beyond our means at the moment.

**Figure 6:**
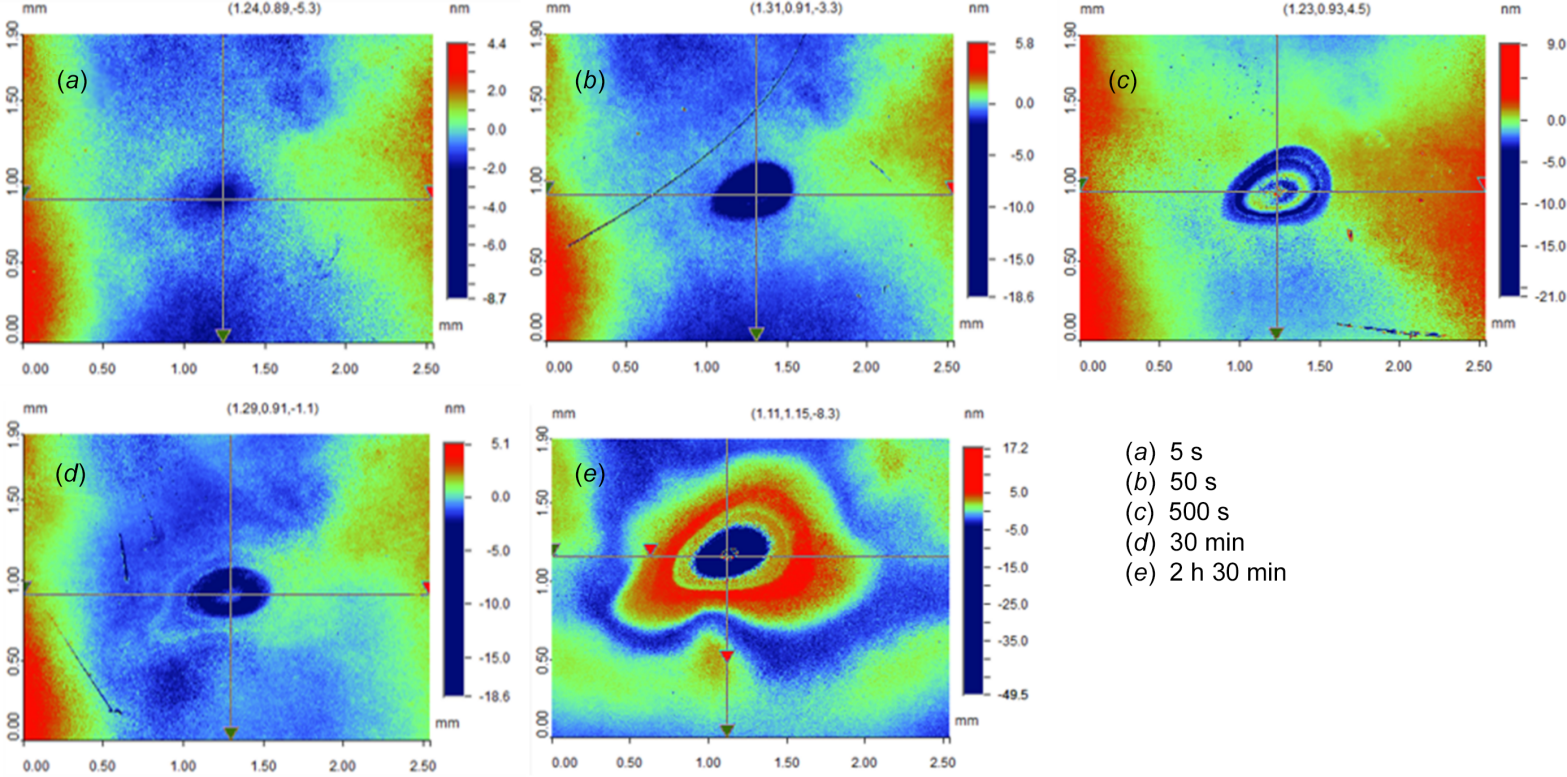
Surface characterization of damage craters on the Au film after 50 µJ FEL irradiation with different exposure times.

Fig. 6 ▸ presents the variation of the crater shape while increasing the time of exposure from 5 s to 2 h 30 min with 50 µJ FEL pulse energy. At 5 s, the crater does not have a rise at its center, and the size of the crater is relatively small compared with the craters from longer exposure times. Starting from 50 s (or somewhere between 5 s and 50 s, to be precise), a rise at the crater center occurs. As the exposure time increases, it is easy to see an increase in the severity of the shockwave effect on the crater shape due to the ultrafast bombardment. This is good evidence that the surface is liquefied during the damaging process as it shows hydro­dynamic behaviors. This is the basic consideration for our theoretical approaches in later sections.

**Figure 7:**
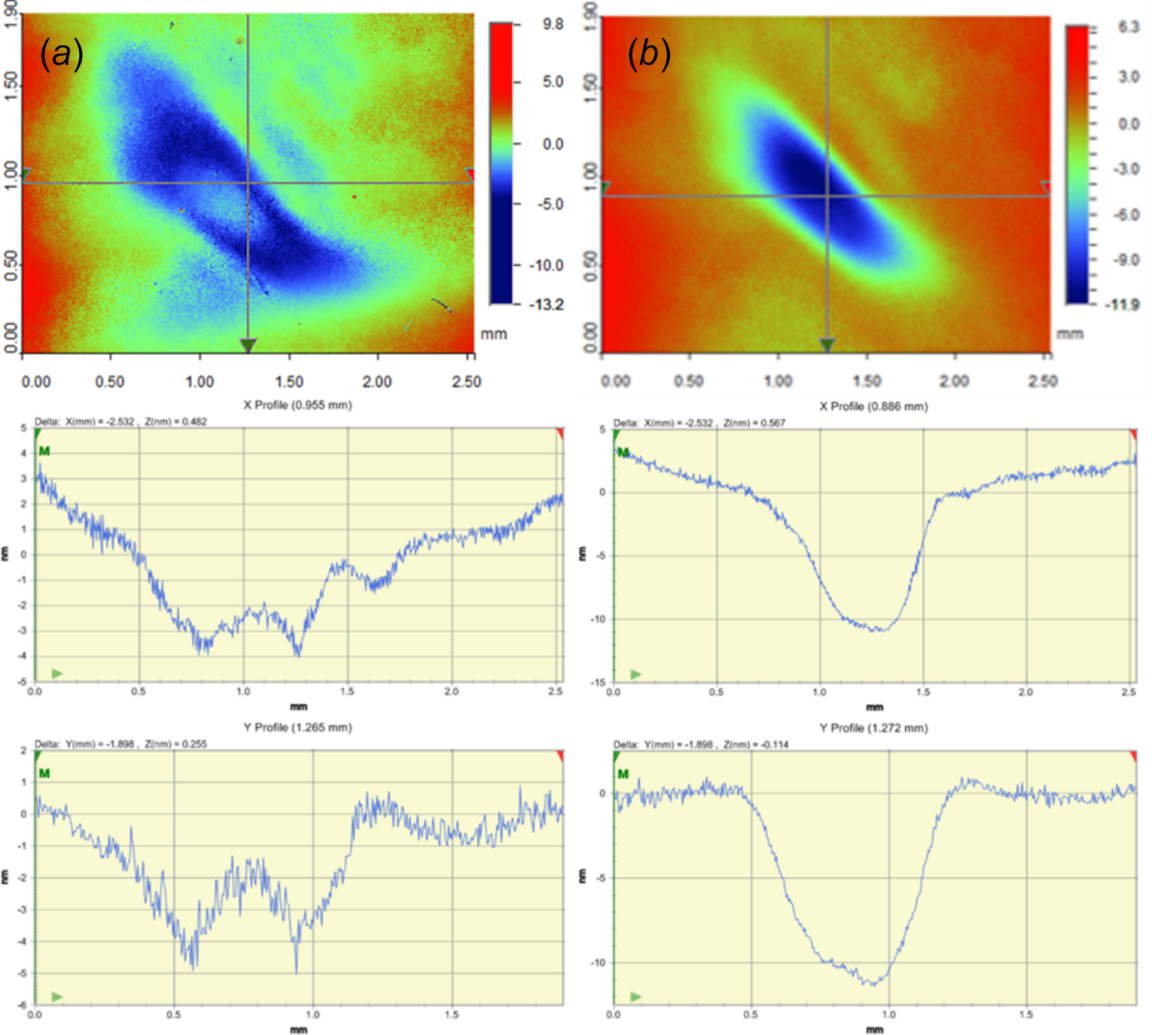
Profilometer-measured surface damage of Au thin film with line profiles along the *x* and *y* axes for exposure times of 2 h 30 min (*a*) and 10 h (*b*) with 5 µJ FEL pulse energy.

In contrast to the 50 µJ cases, for the 5 µJ FEL exposure it is the crater with the shorter exposure time of 2 h 30 min that shows a rise in its center [Fig. 7 ▸(*a*)]. When the exposure time increases to 10 h, the rise in the center seems to have been flattened and smoothened [Fig. 7 ▸(*b*)]. A smaller and shallower ‘side crater’ of the main crater can be observed, showing that the 1st-order and −1st-order diffraction of the FEL also cause damage. This indicates that the damage threshold may even be much lower than that of the 5 µJ case. Comparing Fig. 7 ▸ with Fig. 6 ▸, the shapes of the craters are also different from those of the 50 µJ cases. This is because variation of the incidence FEL pulse energy is realized by manipulating the parameters of the undulators. Consequently, the variation in beam shape appears as a side effect.

**Figure 8:**
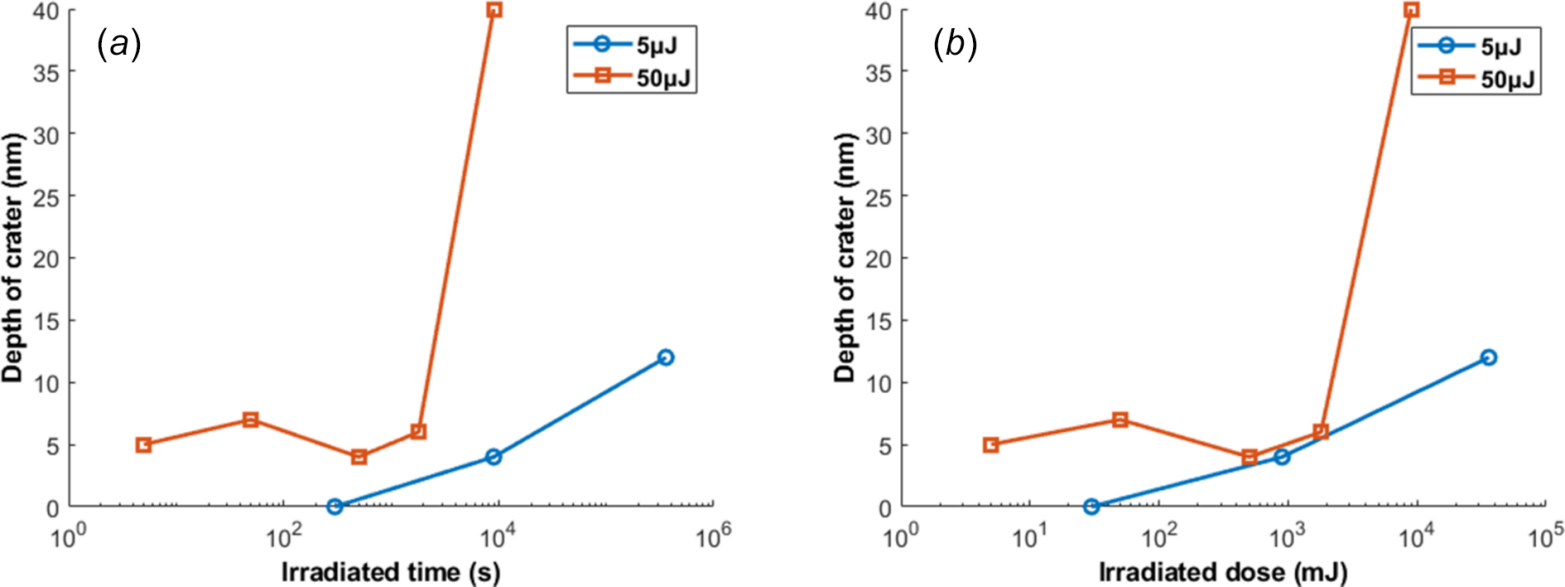
Crater depth versus irradiated time (*a*) and irradiated dose (*b*) for 5 µJ (blue solid line) and 50 µJ (red solid line) FEL pulse energy.

Fig. 8 ▸ presents the crater depth versus exposure time and dose for 5 µJ and 50 µJ FEL pulse energy. The data are summarized in Table 1 ▸. According to the trend, the extent of the surface damage becomes aggravated as the exposure time increases for both cases. For the 50 µJ cases, the crater depth remains around 5 nm for an exposure time from 5 s to 30 min. But the situation deteriorates sharply afterwards, showing a 40 nm crater depth after 2 h 30 min of FEL exposure. For the 5 µJ cases, the trend is rather mitigated. The crater depth smoothly increases from 0 to 12 nm as the exposure time goes from 5 min to 10 h. However, for both FEL pulse energies, saturation is not observed. One may predict further deterioration of the damage if the exposure time is extended. In Fig. 8 ▸, despite similarities in crater depth of the two curves (particularly for the 50 µJ/30 min and 50 µJ/2 h 30 min cases which are comparable dose-wise), the mechanism of damage aggravation may be much more complicated than being linearly proportional to the time of irradiation. Therefore, for the theoretical analysis in the following section, we will focus on the pulse energy instead of dose, which is a more blurred parameter.

## 4. Theoretical approach

The damage of the Au thin film is simulated by using conventional single-pulse damage threshold calculation formulae, a one-dimensional thermal diffusion model and finite-element analysis with *ANSYS* (https://www.ansys.com/).

### 4.1. Calculation of the conventional single-pulse damage threshold

The principle of the conventional calculation method of the single-pulse damage threshold is to compare the irradiation energy received by a single atomic lattice, *W*_atom_, with the melting dose of the lattice, *W*_melt_, to determine whether damage can occur (Sinn *et al.*, 2011 ▸). The basic formulas are as follows,
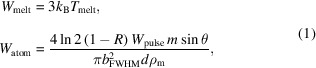
where *k*_B_ is Boltzmann’s constant, *T*_melt_ is the melting point of the material, *R* is the reflectivity of the material, *W*_pulse_ is the incident light power, *m* is the atomic mass, θ is the grazing-incidence angle of the incident light relative to the surface of the optical element, *b*_FWHM_ is the cross section of the incident light spot, *d* is the penetration depth and ρ_m_ is the material density. The condition of the film damage can be determined by comparing the irradiation energy received by a single atomic lattice with the irradiation dose required for the melting of the lattice. That is to say, damage occurs if *W*_atom_ > *W*_melt_. The basic parameters of the material required for the above calculation are obtained from the CXRO database (Henke *et al.*, 1993 ▸; https://cxro.lbl.gov/).

In our case, *W*_atom_ > *W*_melt_ when the single-pulse energy *W*_pulse_ > 233 µJ considering the irradiated surface of 1.2 mm (FWHM). Therefore, in theory there should be no damage for single-pulse energies of 5 µJ and 50 µJ. This is against our observation.

### 4.2. One-dimensional thermal diffusion

In this experiment the size of the light spot is about 1 mm, while the diffusion length in the depth direction (normal incidence) is only tens of nanometres, so the transverse temperature gradient is much smaller than the depth. In this case it is reasonable to use a one-dimensional thermal diffusion model (Sobierajski *et al.*, 2016 ▸) to simulate the heat diffusion in the depth direction of the Au film after a single FEL irradiation. Because the damage of the Au film involves a solid–liquid phase change (the gasification process is ignored here), it is more convenient to describe the whole process with enthalpy. As a metal crystal, Au will continue to absorb heat after the temperature reaches the melting point, and the temperature will not change until it is completely melted. The temperature will not change during this process, but the enthalpy will continue to increase (Eyres *et al.*, 1946 ▸). The increased enthalpy can be expressed by the latent heat of the phase change.

**Figure 9:**
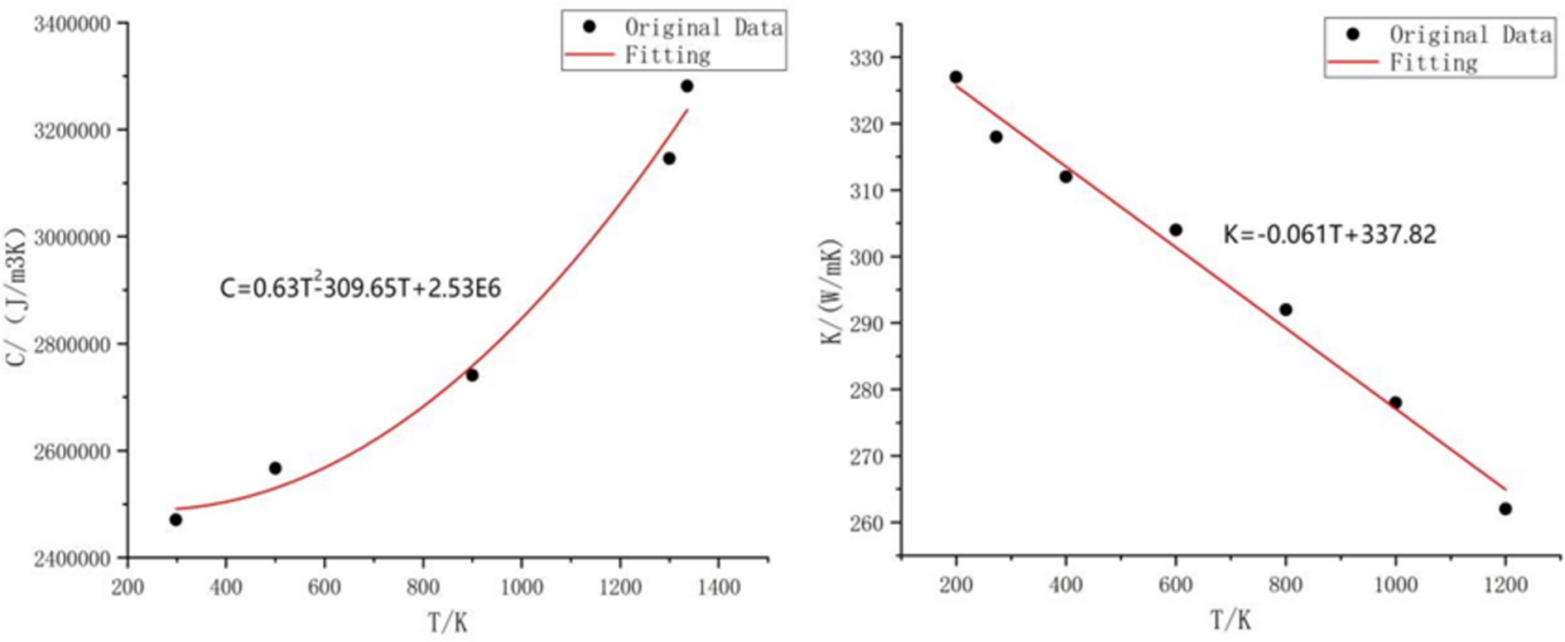
Left: relationship between heat capacity *C* and temperature *T* of Au. Right: relationship between thermal conductivity *K* and temperature *T* of Au.

Therefore, the change of enthalpy in the process of Au film damage can be expressed by the following two formulas,
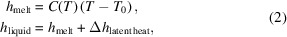
wherein *C*(*T*) is the heat capacity of the material, which is related to the temperature, and *T*_0_ is the reference temperature. In this experiment, *T*_0_ ≃ 298 K [*H*(*T*_0_) = 0]. For one-dimensional thermal diffusion, there are the following differential equations,
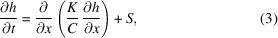
where *h* [J m^−3^] is the enthalpy per unit volume of the sample, *K* [W m^−1^ K^−1^] is the thermal conductivity of the sample, *C* [J km^−3^] is the heat capacity of the sample and *S* [W m^−3^] is the heat source term. Since the thermal conductivity *K* and heat capacity *C* of materials are related to temperature, it is necessary to query the NIST database [thermodynamical parameters were taken from *NIST Chemistry WebBook* (https://webbook.nist.gov/chemistry/)] to obtain the thermal conductivity *K* and heat capacity *C* data in the temperature range 298–1337 K (Au melting point) and fit the functional relationship between *K*(*T*) and *C*(*T*). The data fitting image and function relationship are shown in Fig. 9 ▸. By using the heat capacity data of Au shown in the figure, and the latent heat of the phase change of Au (66.2 J g^−1^), the melting enthalpy of Au and the enthalpy of liquid Au can be calculated to be 2.86 GJ m^−3^ and 4.07 GJ m^−3^, respectively. When the enthalpy of Au is lower than 2.86 GJ m^−3^, the corresponding temperature of the Au film is lower than the melting point. When the enthalpy of Au is higher than 2.86 GJ m^−3^ and lower than 4.07 GJ m^−3^, the temperature of the Au film is kept at the melting point and the melting continues. At this time damage begins to occur. When the enthalpy of Au is higher than 4.07 GJ m^−3^, the heated part of the Au film is completely liquefied.

The heat source term *S* corresponds to the absorption of radiation. Its intensity decays exponentially with depth. This can be described by the Lambert–Beer law (Steen & Mazumder, 2010 ▸),
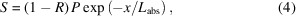
where *R* is the reflectivity of the Au film calculated from the complex refractive index from the CXRO database (Henke *et al.*, 1993 ▸; https://cxro.lbl.gov/), *P* [W m^−2^] is the light source power density and *L*_abs_ [m] is the penetration depth, which is about 13 nm under the experimental conditions.

**Figure 10:**
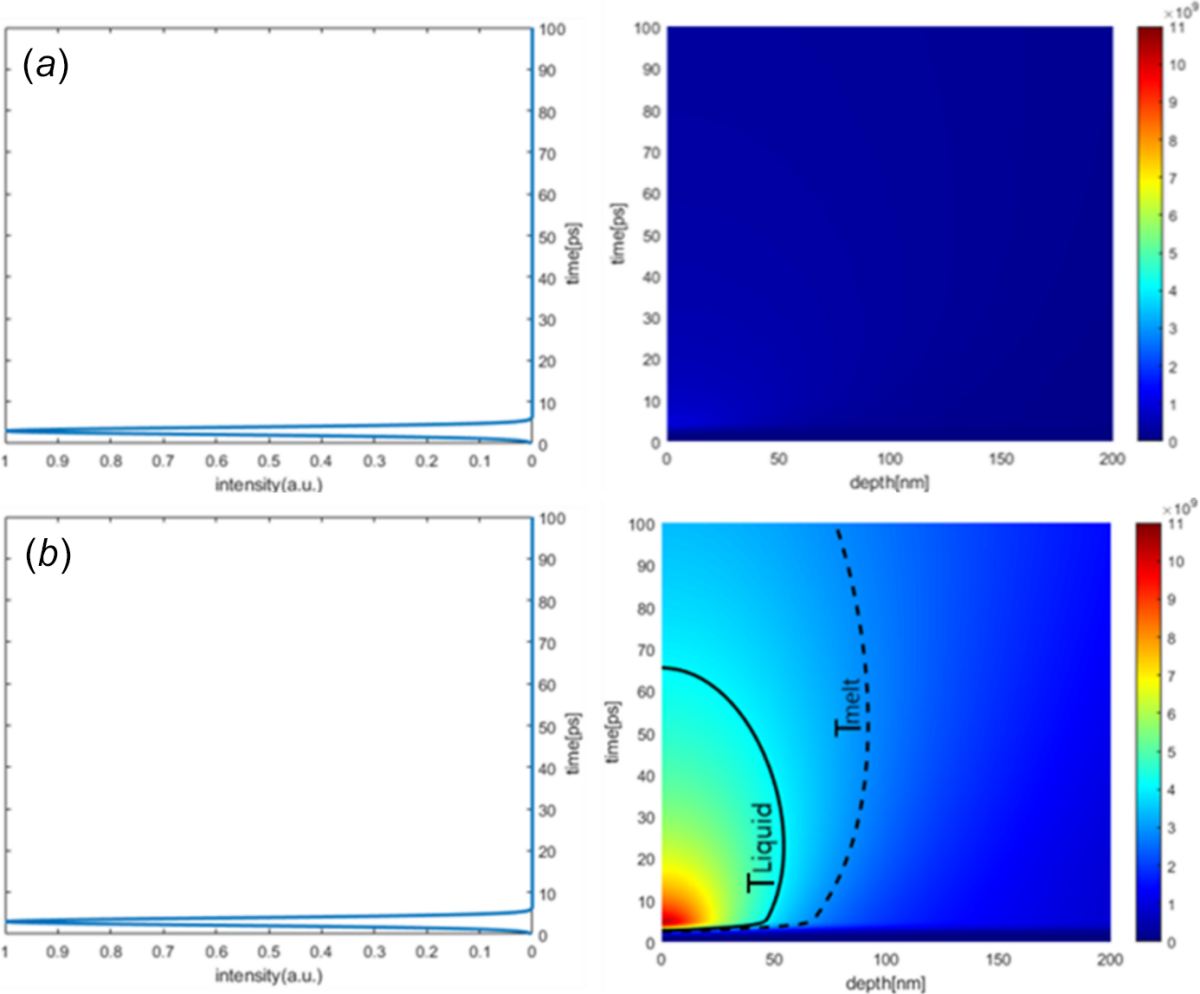
Simulation of one-dimensional thermal diffusion with 5 µJ (*a*) and 50 µJ (*b*) pulse energy of 121.6 nm FEL irradiation on Au thin film. Subfigures on the left present the FEL pulse structure on time scale of the simulation.

One-dimensional thermal diffusion simulation is carried out for FEL pulse energies of 5 µJ and 50 µJ by applying the model mentioned above. The results are presented in Fig. 10 ▸. The areas for local enthalpy of 2.86 GJ m^−3^ (for *T* = *T*_melt_) and 4.07 GJ m^−3^ (for *T* = *T*_liquid_, start of the liquid phase) are marked in the figure using dashed and solid lines, respectively. Since the amount of heat that passes through the Au layer into the Cr layer is extremely small, the Cr layer is neglected in the model to simplify the calculations. As the repetition rate of the FEL beam is merely 20 Hz, which equals 50 ms between two adjacent pulses, heat accumulation will not occur in our case. The intrinsic cooling process, as well as the photoexcited dynamics and relaxation, are expected to be finished. Therefore, in this study, multiple-shot accumulated damage is not considered.

As the calculation mainly focuses on whether damage occurs, the complex case of reflectivity variation due to the topographic modification is not discussed. The FEL pulse length is approximate to the front seed laser pulse length, which is 2 ps (FWHM). The pulse time domain profile is considered as a Gaussian for the calculation.

### 4.3. Finite-element analysis with *ANSYS*

**Figure 11:**
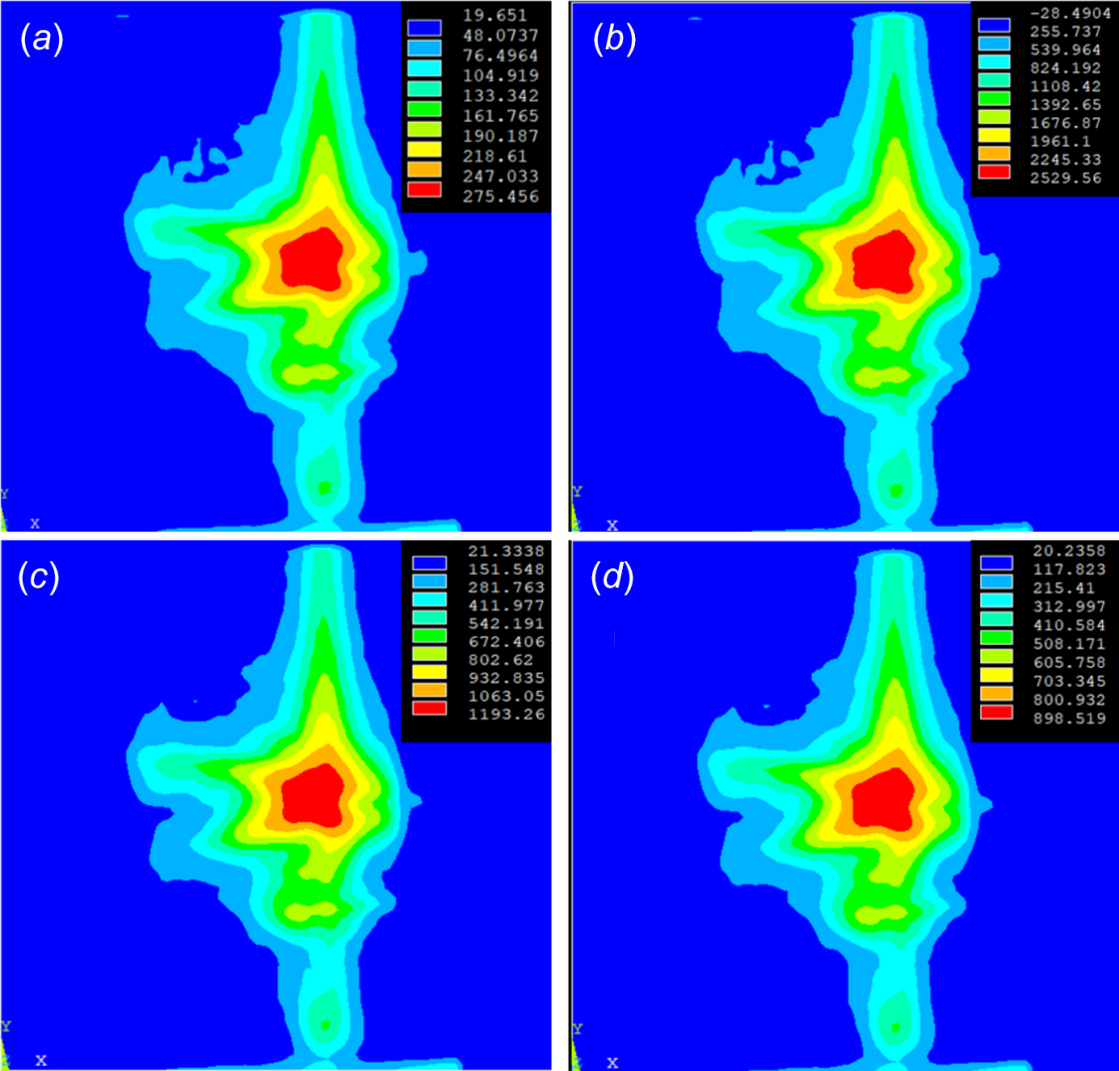
Finite-element analysis of the temperature distribution of the Au film upon FEL exposure. (*a*) Surface with 5 µJ FEL pulse. (*b*) Surface with 50 µJ FEL pulse. (*c*) Depth 16 nm with 50 µJ FEL pulse. (*d*) Depth 20 nm with 50 µJ FEL pulse.

Multilayer elements of *ANSYS* software are applied for the finite-element analysis of the FEL exposure of the sample. In this analysis, we mainly focus on the temperature distribution of the Au film caused by the FEL exposure without considering material removal. The 200 nm Au film is equally decomposed into 50 layers of 4 nm Au, and the Cr adhesion layer is considered to be a 10 × 3 nm multilayer. The beam profile is taken from the CCD measurement (from Fig. 1 ▸) normalized by the pulse energies 5 µJ and 50 µJ individually while considering a single shot of the FEL of pulse length 2 ps (FWHM). Instead of a Gaussian fit, the original profile is used in this case. The result of the analysis is presented in Fig. 11 ▸. With the 5 µJ FEL pulse, the maximum temperature of the surface is only 275°C [Fig. 11 ▸(*a*)], which is far below the Au melting point of 1064°C. Thus, in theory, there should be no damage in this case. This is in line with the previous calculation of 1D thermal diffusion, but is against our experimental observation. One must note that the analysis using *ANSYS* may not be accurate for picosecond phenomena. Although the FEL pulse length of 2 ps is still of the order of magnitude of the phonon thermalization, *ANSYS* analysis is more dedicated for the thermal equilibrium case which is over 10 ps according to the literature (Caruso & Novko, 2022 ▸). With such consideration, it can be expected that the calculated temperature in the Au film is underestimated, which in return explains the mismatch between the calculations and the experimental results. Another contribution of such a mismatch could be the fact that the YAG+CCD spot size determination means lacks accuracy, thus the irradiated surface temperature is overestimated. For the 50 µJ FEL pulse, the surface temperature of the Au film reaches up to 2260°C [Fig. 11 ▸(*b*)] and causes surface damage due to the melting of Au.

## 5. Conclusion and outlook

In this paper, we evaluate the damage threshold of an Au-coated flat mirror upon UV FEL irradiation. According to the experimental result, the single-shot damage threshold is expected to be lower than 2 mJ cm^−2^, which relates to a 5 µJ FEL pulse energy considering a 1.2 mm (FWHM) spot. This value differs from the one that is predicted by multiple theoretical approaches, including conventional single-pulse damage threshold calculation formulae, one-dimensional thermal diffusion model and finite-element analysis with *ANSYS*. Conventional SSDT calculation predicts that no damage should occur, while the thermal diffusion model and finite-element analysis indicate that damage should occur with the FEL pulse energy somewhere between the two selected values of 5 µJ and 50 µJ. As the stability of the FEL pulse energy is not satisfactory, the accuracy of such a result is limited to the order of magnitude of the incidence pulse energy. Considering the fact that a damage crater is observed for the diffraction pattern of the FEL beam on the Au surface, the actual SSDT is expected to be even lower. The damage does not exhibit saturation. Therefore, further deterioration of the damage is expected to occur if the FEL exposure time is extended.

Grazing-incidence geometry can be considered to reduce the absorption of the FEL on the Au coating and increase the surface of the irradiated area. Mathematically this leads to a lower energy density for the exposure. The SSDT in this case can be converted and compared with the normal-incidence case. Furthermore, a photodiode can be placed after the sample mirror to monitor the reflectivity of the mirror throughout the entire FEL exposure process. In theory, by synchronizing the readings of such a photodiode with the data from an intensity monitor installed upstream of the beamline, one may obtain accurate reflectivity of the mirror for each pulse.

There may be another reason for the mismatch between the experimental results: the selection of beam spot characterization. Limited by the YAG resolution and sensitivity, it is likely that the detailed shape of the spot is missed (Manfredda *et al.*, 2022 ▸). The scintillating area can be blurred and enlarged compared with the FEL beam cross-section. This may lead to the fact that the irradiated surface is actually smaller than that measured by the CCD camera. In future experiments a pinhole of micrometre size can be considered, together with a photodiode mounted right after it, to probe the shape and intensity distribution of the millimetre-sized beam.

## Figures and Tables

**Table 1:** Overview of the damage characterization

Irradiated time (s)	Irradiated dose (mJ)	Crater depth (nm)
5 µJ pulse energy		
300	30	0
9000	900	4
360000	36000	12

50 µJ pulse energy		
5	5	5
50	50	7
500	500	4
1800	1800	6
9000	9000	40
